# A Novel Single-Nucleotide Polymorphism in *WNT4* Promoter Affects Its Transcription and Response to FSH in Chicken Follicles

**DOI:** 10.3390/genes13101774

**Published:** 2022-10-01

**Authors:** Conghao Zhong, Yiya Wang, Cuiping Liu, Yunliang Jiang, Li Kang

**Affiliations:** 1 Shandong Provincial Key Laboratory of Animal Biotechnology and Disease Control and Prevention, College of Animal Science and Veterinary Medicine, Shandong Agricultural University, Tai’an 271018, China; 2 College of Life Science, Qilu Normal University, Jinan 250200, China; 3 Qishan Animal Husbandry and Veterinary Station, Zhaoyuan 265413, China

**Keywords:** chicken, *WNT4*, SNP, FSH, production traits

## Abstract

The signaling pathway of the wingless-type mouse mammary tumor virus integration site (Wnt) plays an important role in ovarian and follicular development. In our previous study, *WNT4* was shown to be involved in the selection and development of chicken follicles by upregulating the expression of follicle-stimulating hormone receptors (*FSHR*), stimulating the proliferation of follicular granulosa cells, and increasing the secretion of steroidal hormones. FSH also stimulates the expression of *WNT4*. To further explore the molecular mechanism by which FSH upregulates *WNT4* and characterize the cis-elements regulating *WNT4* transcription, in this study, we determined the critical regulatory regions affecting chicken *WNT4* transcription. We then identified a single-nucleotide polymorphism (SNP) in this region, and finally analyzed the associations of the SNP with chicken production traits. The results showed that the 5′ regulatory region from −3354 to −2689 of *WNT4* had the strongest activity and greatest response to FSH stimulation, and we identified one SNP site in this segment, −3015 (G > C), as affecting the binding of NFAT5 (nuclear factor of activated T cells 5) and respones to FSH stimulation. When G was replaced with C at this site, it eliminated the NFAT5 binding. The mRNA level of *WNT4* in small yellow follicles of chickens with genotype GG was significantly higher than that of the other two genotypes. Moreover, this locus was found to be significantly associated with comb length in hens. Individuals with the genotype CC had longer combs. Collectively, these data suggested that SNP−3015 (G > C) is involved in the regulation of *WNT4* gene expression by responding FSH and affecting the binding of NFAT5 and that it is associated with chicken comb length. The current results provide a reference for further revealing the response mechanism between WNT and FSH.

## 1. Introduction

As a conservative pathway, Wnt signaling regulates multiple developmental processes and occurrences of disease, such as stem cell self-renewal, cell proliferation, cell fate determination and early embryonic development and differentiation [1,2,3]. The members of the Wnt family are a class of secreted glycoprotein signaling molecules with localized action that is generally used as ligands to participate in signal transduction. At least 15 receptors or co-receptors such as the Frizzled (Fzd) protein family are recognized and bound by Wnt ligands [4,5]. Wnt ligands use two signaling pathways, β-catenin-dependent (canonical) and β-catenin-independent (non-canonical) [6,7]. The most widely understood is the classical Wnt/β-catenin signaling pathway, which regulates gene transcription through β-catenin entering the nucleus. The Wnt signaling pathway is essential for normal ovarian and follicle development. Wnt family members can regulate many physiological processes during ovarian growth and development, such as follicular development, ovulation and luteinization [8,9].

Some Wnts and their homologous receptor components are expressed in postnatal ovaries, but their role in ovarian physiology remains unclear. Many studies have proven that *WNT4* serves an important regulatory function in adult ovarian follicles. The overexpression of *WNT4* in the granulosa cells of eCG-treated mice upregulates the expression of β-catenin and the key genes *CYP11A1*, *CYP19A1* and *StAR* in the synthesis of gonadal steroid hormones [8]. In chickens, *WNT4* affects the growth, differentiation and development of fallopian tubes and is regulated by estrogen [10,11]. Our previous study revealed that the expression of *WNT4* was the highest in the granulosa cells of the small yellow follicles in chickens and was lower in the hierarchal follicles. *WNT4* upregulates the expression of *FSHR* and downregulates the expression of *AMH* and *OCLN*, promotes the expression of *StAR* and *CYP11A1*, and stimulates the proliferation of granulosa cells. Additionally, *WNT4* expression was stimulated by FSH. The interaction of *WNT4* and FSH signaling strengthened from the prehierarchical to the hierarchical follicles during the process of follicle selection [12]. FSH is a critical hormone that regulates chicken follicle selection, and *WNT4* also participates in this process by regulating the expression of *FSHR*. However, the molecular mechanism by which FSH upregulates *WNT4* expression is still unclear.

In the current study, we investigated the regulatory mechanism of chicken *WNT4* transcription. First, we determined the critical regulatory cis-elements responsible for *WNT4* transcription that are also responsive to FSH treatment. We identified two single-nucleotide polymorphisms (SNPs) in the 5′ regulatory region of the chicken *WNT4* gene and analyzed their associations with production traits in hens. Finally, we analyzed the mechanism of SNP that is associated with chicken comb length. These results may help us to further explore the molecular mechanism by which FSH upregulates *WNT4* expression. This study also laid a foundation for screening potential DNA markers in the molecular breeding of chickens for egg laying.

## 2. Materials and Methods

### 2.1. Animals and Sample Collections

For this study, we used three breeds of hen, Hy-line brown, Jining Bairi and Sunzhi, with different production performances (Jining Bairi hens have smaller age of first egg. Hy-line brown hens are widely cultivated commercial chickens with stable production performance. Sunzhi hens are both meat and egg breed.). The hens were randomly selected from the local farm affiliated with Shandong Agricultural University. The egg- laying traits of the Jining Bairi population were recorded individually for use in association analysis. All chickens had free access to water and feed. The chickens were housed in separate cages with a daily light period of 16 h, and egg laying was monitored to determine the timing and regularity of laying. Genomic DNA was extracted from blood samples collected from the wing vein using a DNA extraction mini-kit (Tiangen Biotech, Beijing, China). All sampled hens were killed by cervical dislocation immediately after oviposition, and their abdominal cavity was opened. Preovulatory follicles were carefully collected from the laying hens and placed in phosphate-buffered saline (PBS) with 1% penicillin/streptomycin for cell culture [13]. All of the animal experiments were approved by the Institutional Animal Care and Use Ethics Committee of Shandong Agricultural University and performed in accordance with the “Guidelines for Experimental Animals” of the Ministry of Science and Technology of China.

### 2.2. Cell Culture

The hierarchical follicles were isolated from the egg-laying hens and placed in PBS. The yolks of the follicles were carefully removed with ophthalmic forceps. The granulosa cells (GCs) were isolated from the hierarchical follicles and then dispersed by treatment with 0.25% trypsin-EDTA (Gibco, Camarillo, CA, USA) at 37 °C for 10 min with gentle oscillation in a centrifuge tube. After centrifugation, the GCs were suspended in a culture medium (M199 with 10% fetal bovine serum and 1% penicillin/streptomycin) and subsequently seeded in 24-well culture plates at a density of 1 × 10^5^/well. The number of cells was detected using Trypan blue. Cells were cultured at 38 °C in an atmosphere of water-saturated 5% CO_2_ for 24 h.

### 2.3. Construction of WNT4 Promoter Deletion Vectors and Site-Directed Mutation

The region from −3354 to +252 bp in the 5′-regulatory region of the chicken *WNT4* promoter, as well as four promoter deletion fragments, −2689/+252, −1875/+252, −1188/+252 and −535/+252, were amplified from the hen genomic DNA, where +1 was the transcription initiation site, the five promoter fragments were designed according to the chicken *WNT4* gene sequence (NC_052552.1). Five forward primers contained the KpnI site at the ends, and one reverse primer located downstream of the transcription start site contained the HindIII site at the ends (the primer sequences are listed in Table 1). All PCR fragments were digested with KpnI and HindIII restriction enzymes and ligated with pGL3-Basic vector (Promega, Madison, WI, USA).

Two plasmids including the wild type (pGL3-G) and the mutation type (pGL3-C) were constructed to assess the functionality of this transcription factor binding site in the *WNT4* promoter. The primers for the g.−3015 (G > C) mutations were designed using the −3354 to −2689 region of the *WNT4* gene promoter as the template (the primer sequences are listed in Table 1). The PCR products were digested with Dpn1 methylase, so the plasmid template to be mutated could be removed and the plasmid containing the mutation site could be retained.

### 2.4. Cell Transfection and Luciferase Assay

GCs were plated on 24-well plates for transient transfection experiments using Lipofectamine LTX and Plus Reagent (Invitrogen). The cells were transfected with pGL4.74 control vector (Promega, Madison, WI, USA), and the five *WNT4* luciferase plasmids, which differed in length (800 ng/well), were added to the wells 6 h after transfection along with recombinant FSH. In another experiment, the pGL4.74 control vector (Promega, Madison, WI, USA), the wild-type plasmid and the mutation-type plasmid (800 ng/well) were used to transfect cultured GCs with recombinant FSH. Twenty-four hours after transfection, these cells were lysed for a luciferase activity assay.

Luciferase activity was measured using the Dual-Luciferase Reporter Assay System according to the manufacturer (Promega, Madison, WI, USA). The enzymatic activity of luciferase was measured with a luminometer (Modulus TM, Turner Biosystems). The individual values were averaged for each experiment, and the transfections were performed in at least triplicate. Empty pGL3-basic was used as the control. Luciferase activity was calculated by dividing the Firefly luciferase activity by the Renilla luciferase activity.

### 2.5. SNP Identification, Polymorphism and Association Analysis

We used 50 Jining Bairi, Hy-line Brown and Sunzhi hens as templates for PCR amplification to the critical promoter region of *WNT4* (−3353 to −2689), and then sequenced the amplifications. We analyzed the data, sequenced bidirectionally, using the DNAMAN program (version 7.212, Lynnon Corp., Quebec, Canada) to determine the potential SNPs within these amplifications. The primer pairs amplified the −3353 to −2689 fragments are shown in Table 1. The genotypes at the −3015 SNP site were determined by Kompetitive Allele Specific PCR (KASP) (Baygene Biotechnology Co., Shanghai, China) in the Jining Bairi population. The genotype and allele frequencies and the Hardy-Weinberg equilibrium *p*-value were calculated using the Tools for Population Genetic Analyses software (http://www.marksgeneticsoftware.net/tfpga.htm, accessed on 3 March 2019). The associations of SNPs with production traits in the Jining Bairi population were analyzed using the following general model in SPSS (SPSS Inc., Chicago, IL, USA): Y_ij_ =*μ*+ G_i_ + *e_ij_*, where Y_ij_ is the phenotypic value of traits, *μ* is the population mean, G_i_ is the fixed effect of genotype and *e_ij_* is the random error effect.

### 2.6. Electrophoretic Mobility Shift Assay (EMSA)

Genomatix software (www.genomatix.de) was used to predict that −3015 (G > C) would be the transcription factor binding sites of NFAT5 that regulates the transcription of *WNT4* by responding to FSH. For further verification, HIH3T3 cells were seeded at a density of 1 × 10^6^ cells/mL and incubated in DMEM with 10% FBS at 37 °C for 72 h. The nuclear extracts prepared from the cells were incubated with biotin-labeled double-stranded oligonucleotides containing the consensus sequences for NFAT5 (5′-TTTATCCCAgGGAAACCTTCACAGTGCATTC-3′) for an additional 4 h. GATA1 (5′-CACTTGATAACAGAAAGTGATAACTCT-3′) was used as a control. EMSA was performed using Non-Radioactive EMSA Kits with Biotin-Probes (Viagene, Tampa, FL, USA). The DNA-protein complex and unbound probe were electrophoresed on a 6% native polyacrylamide gel and visualized with Western blotting. The NFAT5 monoclonal antibody was used for the super shift, and standard NFAT5 was used as a positive control.

### 2.7. Real-Time Quantitative PCR

We further verified the effect of −3015 (G > C) mutation on *WNT4* gene expression by real-time quantitative PCR (qPCR). The total RNA was extracted from chicken small yellow follicles (6-8 mm) of 15 individuals with different genotypes using TRIzol reagent (Invitrogen). The quality of the total RNA samples was tested by gel electrophoresis and spectrophotometry. Synthesis of the cDNA was performed using a PrimeScript RT Reagent Kit with a gDNA Eraser (TaKaRa, Dalian, China) according to the manufacturer’s protocol. qPCR of the mRNA expression level of *WNT4* was performed using an SYBR Premix Ex Taq™ II kit (TaKaRa, Dalian, China) on a Light Cycler 480 real-time PCR system (Roche, Basel, Switzerland) as follows: 95 °C for 30 s, followed by 40 cycles of denaturation at 95 °C for 10 s and annealing and extension at 58 °C for 20 s. The primers were designed according to the chicken *WNT4* mRNA sequence (NM_204783.2) and internal reference gene *GAPDH* mRNA sequence (NM_204305.2), the primer sequences are listed in Table 1. Melting curves were used to confirm the specificity of each product, and quantitative analysis of the data was performed using the 2^−ΔΔCT^ relative quantification method.

### 2.8. Statistical Analyses

The experiments were performed a minimum of three times using tissues from different hens. All data are presented as the means ± SEM. The differences between groups were determined by one-way ANOVA followed by Duncan’s test in SPSS (SPSS Inc., Chicago, IL, USA). The differences between groups were considered statistically significant when *p* < 0.05.

## 3. Results

### 3.1. Critical Region of WNT4 Gene Response to FSH in Chicken GCs

**Figure 1:**
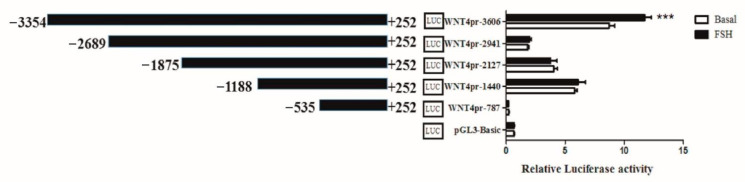
Luciferase assay of the effects of FSH on *WNT4* promoter activity in chicken GCs. The numbering refers to the transcription initiation site, designated as +1. Each bar represents the means ± SEM. *** indicates *p* ≤ 0.001.

As *WNT4* plays an essential role in follicle selection, and FSH could regulate its expression, we set out to analyze the mechanisms regulating *WNT4* transcription. The luciferase activity assay on chicken preovulatory follicle GCs transfected with different *WNT4* promoter vectors (Table 1) showed that deletion from −3354 to −2689 greatly decreased luciferase activity, indicating that positive regulatory elements exist in this region. Additionally, the region from −3354 to −2689 had the strongest response to FSH (10 ng/mL) stimulation (Figure 1), suggesting that this region may contain cis-acting response elements to FSH to regulate the transcription of the chicken *WNT4* gene. These findings demonstrated that when regulating the expression of *WNT4*, FSH is closely related to the region from −3354 to −2689 in the *WNT4* promoter.

### 3.2. Polymorphisms in the Critical Promoter Region of the Chicken WNT4 Gene

**Figure 2:**
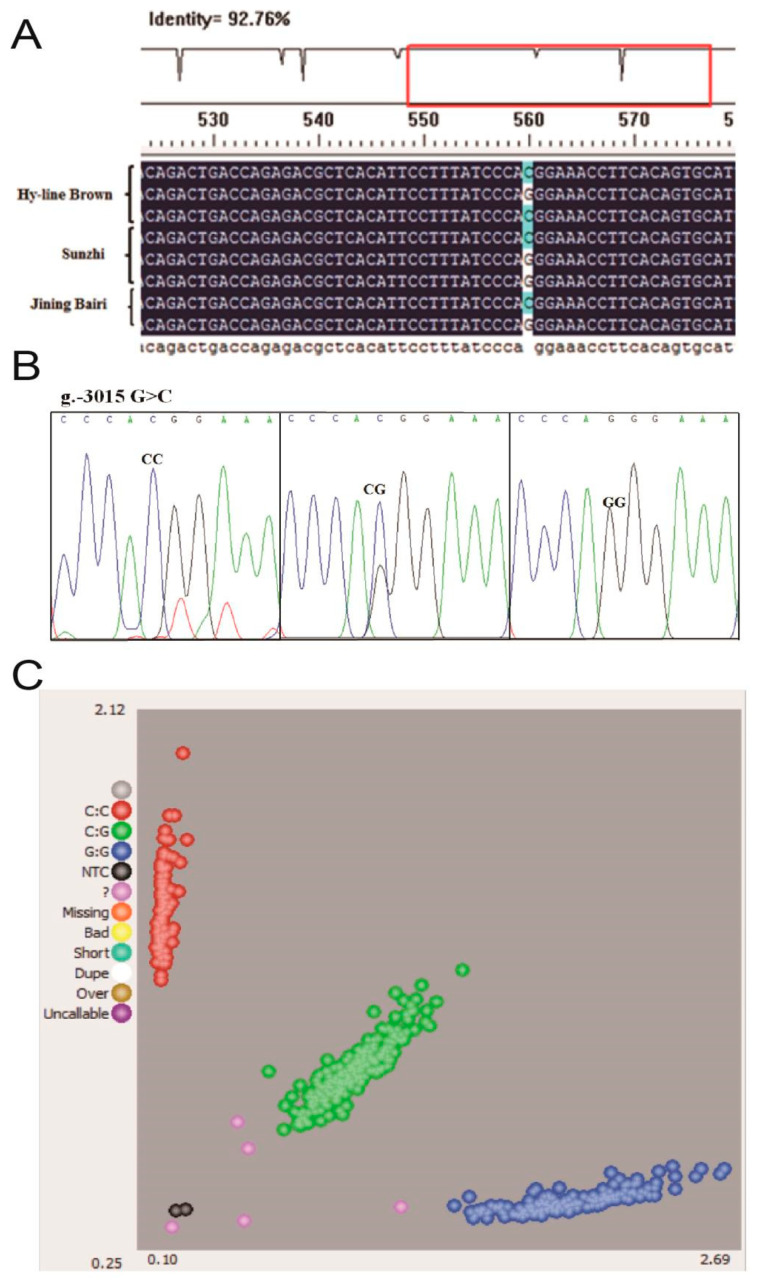
All schemes follow the same formatting. Polymorphisms in the critical promoter region of chicken *WNT4* gene. (**A**) Sequencing alignment of SNP site in Hy-line Brown hens, Jining Bairi hens and Sunzhi hens; one SNP (g.−3015) was detected. (**B**) Genotyping of the SNP (g.−3015) site and three genotypes. (**C**) Genotyping in the Jining Bairi population (*n* = 539) using the KASP method. The number of successful genotyping individuals was 539. NTC (black point), no template controls. These are wells that do not contain any template DNA and will therefore not generate any fluorescent signal. Unused (pink point), samples that have not been assigned a genotyping call because they did not generate a consistent signal or failed to amplify.

The sequence alignment between the promoter regions of the chicken *WNT4* gene in the Hy-line Brown, Jining Bairi and Sunzhi hens showed that the critical promoter region contains an SNP (G > C) at −3015 (Figure 2A). The peak map of polymorphic sites was genotyped using the Chromas software. This polymorphic site has three genotypes (Figure 2B), and the number distribution of the different genotypes is shown in Figure 2C. The number of Jining Bairi population (*n* = 539) represents the samples which are successfully genotyped. According to the results of KASP, the genotype and allele frequency of this SNP locus were calculated (Table 2). At this SNP site, allele G was predominant in the Jining Bairi chicken population.

### 3.3. The Association of SNP−3015 (G > C) of WNT4 Gene with Chicken Production Traits

The statistical analysis is based on the genotype results of the Jining Bairi chicken population (*n* = 539) with production records. Table 3 shows the associations between the genotype of each individual and the egg-laying traits. The results indicate that chickens with genotype CC had a longer comb (*p* < 0.05), and the chickens with genotype CG had more eggs at 50 weeks of age, although the difference was not significant (*p* = 0.075).

### 3.4. The Effects of SNP−3015 (G > C) on the Promoter Activity of the Chicken WNT4 Gene

**Figure 3:**
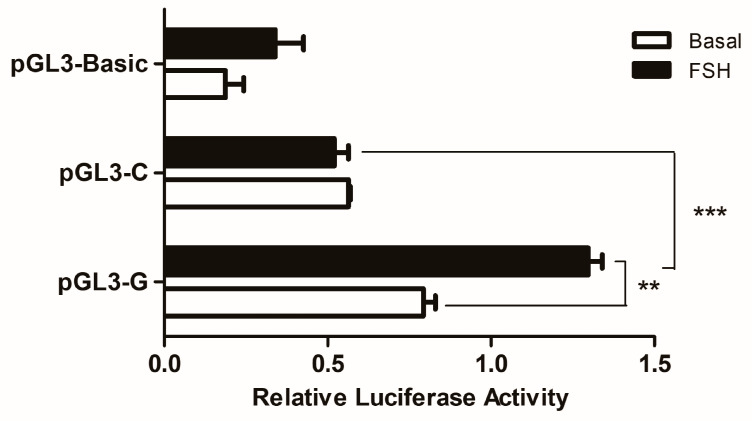
Effects of the SNP on chicken *WNT4* gene transcriptional activity in GCs. It used the dual-luciferase report assay to verify the effects of the SNP (g.−3015) on different genotypes. Each bar represents the means ± SEM. *** indicates *p* ≤ 0.001. ** indicates *p* < 0.01.

Luciferase reporter constructs of pGL3-G and pGL3-C were transiently transfected into GCs to assess whether the SNP could change the effect of *WNT4* gene transcription. As shown in Figure 3, this SNP significantly affected the promoter activity: the promoter with allele G had higher luciferase activity than the promoter with allele C (*p* < 0.001). After FSH treatment, the luciferase activity of the promoter with allele G was significantly (*p* < 0.01) increased than before, as well as still significantly (*p* < 0.01) higher than the activity with allele C.

### 3.5. SNP−3015 (G > C) Affects NFAT5 Binding in Chicken WNT4 Promoter

**Figure 4:**
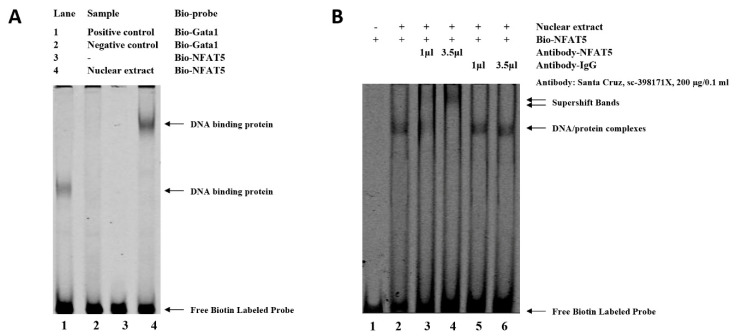
Electrophoretic mobility shift assay (EMSA) of the transcription factor binding sites at the SNP (g.−3015) in chicken *WNT4* promoter. (**A**) EMSA analysis was conducted with biotin-labeled probe in the SNP (g.−3015). Lane 1 and 2, positive or negative control. Lane 3 and 4, labeled probe without or with nuclear extract. (**B**) Super shift assay with NFAT5 monoclonal antibody appeared to correspond to the DNA-protein-antibody complex. Lane 1, labeled probe without nuclear extract. Lane 2, labeled probe with nuclear extract. Lane 3 and 4, labeled probe with nuclear extract + low or high dose of specific antibody (NFAT5). Lane 5 and 6, labeled probe with nuclear extract + low or high dose of nonspecific antibody (IgG).

Analysis with Genomatix revealed that SNP (G > C) at −3015 may affect the binding site of NFAT5, which may be related to the regulation of *WNT4* responses to FSH. An electrophoretic mobility shift assay (EMSA) was performed to identify this transcription factor. As shown in Figure 4, binding to the oligo nucleotide (NFAT5) was detected when the SNP site was G (Figure 4A). While there is a characteristic shift caused by specific protein binding to target nucleic acid, a relative change in mobility does not identify the bound protein in a shifted complex. Then, the identification of the protein bound to the probe was accomplished by including an antibody that is specific for the putative DNA-binding in the binding reaction. As shown in lane 4 of Figure 4B, the NFAT5 antibody binding to the DNA-protein complex will decrease its mobility relative to unbound DNA. The super shift assay with the NFAT5 monoclonal antibody appeared to correspond to the DNA-protein-antibody complex, which further demonstrated that the SNP (g.−3015) was located in the binding site of NFAT5 (Figure 4B).

### 3.6. The Expression Level of WNT4 mRNA in Different Genotype Chicken Individuals

**Figure 5:**
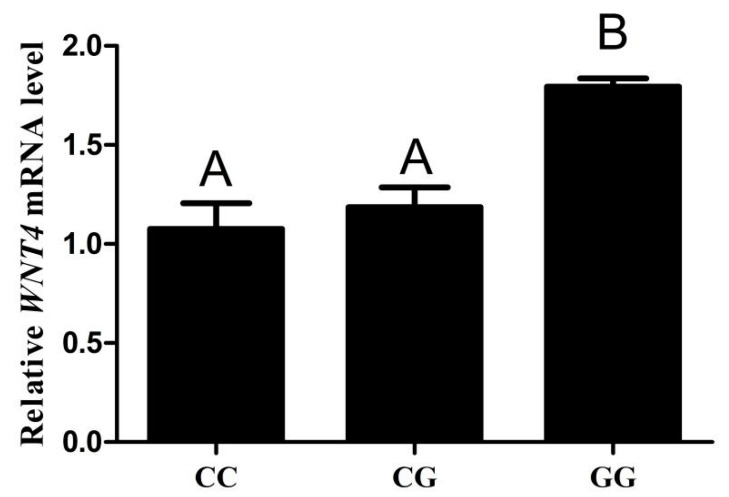
qPCR analysis the mRNA levels of *WNT4* in chicken small yellow follicles with genotypes CC, CG and GG (*n* = 5). Each bar represents the means ± SEM. Bars with different superscript capital letters (A, B) indicate statistically significant differences at *p* < 0.01.

To analyze the effect of SNP (g.−3015) on *WNT4* expression, we examined the mRNA level of *WNT4* in chicken follicles of different genotypes. The results showed that *WNT4* mRNA levels were detected in both the CC, CG and GG chicken small yellow follicles. The mRNA levels from the GG genotype were higher than that from the CC and CG genotypes (*p* < 0.01), but there was no significant difference between the CC and CG genotypes (Figure 5).

## 4. Discussion

In recent years, researchers have made considerable progress in determining the roles of Wnt family members in mammalian ovarian and follicular development. The expression of Wnt family members and their downstream signal elements have been detected in the different grade follicles and corpus luteum of rats, mice, humans and cattle, and they also play a certain regulatory role in follicular development [14,15,16,17,18,19]. *WNT4*, a member of the Wnt family, also plays a key regulatory role in the development and differentiation of the female reproductive system [8,20]. Our previous work has proven that *WNT4* plays a significant role in ovarian follicle selection in chickens, and FSH treatment significantly increased the expression of *WNT4* in GCs [12]. In cows, *WNT4* can enhance the stimulation of FSH during the follicular dominance phase [19]. These results suggest that there is some interaction between *WNT4* and FSH. However, the molecular mechanism of the interaction between them has not been investigated. In our study, we investigated the critical regulatory regions affecting *WNT4* transcription and then identified a single-nucleotide polymorphism (SNP) in this region, in order to provide a reference for further elucidating the regulatory relationship between *WNT4* and FSH.

We found that the 5′ regulatory region from −3354 to −2689 of *WNT4* was the key regulatory region that responded to FSH stimulation and affected chicken *WNT4* gene transcription. We identified SNP−3015 (G > C) in this region as the working site. Two studies showed that the SNPs of the *WNT4* gene can affect its expression and function. SNP rs7521902 in *WNT4* is significantly correlated with the pathogenesis of endometriosis [21], and rs2072920 in *WNT4* is associated with body mass index (BMI) variations in the Han Chinese population [22]. We identified the critical regions of the *WNT4* promoter that contained SNP−3015 (G > C), and we found that SNP−3015 (G > C) was significantly associated with comb length in chickens. A recent study has shown that the Wnt signaling pathway is related to comb growth and development in chickens [23]. Comb size is an important criterion for hen development and egg production [24,25]. Here, the individuals with the CC genotype had longer combs at the −3015 (G > C) mutation site, suggesting a potential marker for improving comb characteristics.

We investigated the effect of SNP−3015 (G > C) on the promoter activity and expression of the chicken *WNT4* gene, and the results showed that this SNP significantly affected the promoter activity. The promoter with allele G had higher luciferase activity and mRNA levels than the promoter with allele C, and allele G responded more actively to FSH stimulation. The transcription factor prediction and EMSA experiment showed that SNP−3015 (G > C) in this key regulatory region was the binding site of the transcription factor NFAT5. When this site is G, it has binding activity. NFAT5 is a transcription factor that participates in many biological processes, including cell differentiation, cellular migration and embryonic development [26,27,28]. Mice lacking NFAT5 have dramatically reduced embryonic viability and significant perinatal lethality due to kidney defects and impaired cardiac development and function [29,30,31]. Many studies have proven the relationship between NFAT and Wnt, as a member of the NFAT family, NFAT5 is also a regulator of Wnt signaling [32,33]. In humans, NFAT5 inhibits β-catenin signaling and participates in the regulation of intestinal cell differentiation [34]. In mice, NFAT5 plays a crucial role in GC proliferation and enhances the Wnt pathway via upregulating the expression of β-catenin and Bcl-2 [35]. Combined with the above research results, we consider that SNP−3015 (G > C) may regulate *WNT4* expression through NFAT5 binding, in turn affecting the Wnt signaling pathway and chicken follicle selection. However, although NFAT5 binding may be involved in the regulation of *WNT4* expression, it does not exclude that the expression of *WNT4* may be regulated by other unknown factors, and the regulation of FSH may also be related to these unknown factors. About this possibility, further in-depth investigation is needed in our next studies.

## 5. Conclusions

In summary, SNP−3015 (G > C) in the *WNT4* promoter region responded to FSH stimulation and appeared to be regulated by NFAT5. The SNP was found to be significantly associated with chicken comb length. These data provide a reference for the further elucidation of the relationship between FSH and *WNT4* in chicken follicles.

## Figures and Tables

**Table 1:** Primers used in this study.

Primer Name	Primer Sequence (5′-3′)
pGL3-WNT4(−3354–+252) (F)	CGGGGTACCGCCTGGAGGTATAATAAGCA
pGL3-WNT4(−2689–+252) (F)	CGGGGTACCTTAGGTGACGGCACAGCA
pGL3-WNT4(−1875–+252) (F)	CGGGGTACCCCAGGGGCATCAGAAT
pGL3-WNT4(−1188–+252) (F)	CGGGGTACCTGCGAGCAGGAAAATGGA
pGL3-WNT4(−535–+252) (F)	CGGGGTACCGTCTACCAGCAAGAGCAGG
Pro-WNT4-1R2	CCCAAGCTTAGAAGGTGGCGAGGATG
WNT4-mut-F	CACATTCCTTTATCCCACGGAAACCTTCACAGTGCA
WNT4-mut-R	TGCACTGTGAAGGTTTCCGTGGGATAAAGGAATGTG
GAPDH-F	GAGGGTAGTGAAGGCTGCTG
GAPDH-R	CACAACACGGTTGCTGTATC
WNT4-F	CCTGTCTTTGGCAAGGTGG
WNT4-R	CATAGGCAATGTTATCGGAGC

**Table 2:** Genotype and allele frequencies at the SNP site of the *WNT4* gene in the Jining Bairi chicken.

Site	Breed (Number)	Genotype Frequency			Allele Frequency		HWE (*p*-Value)
g.−3015	Jining Bairi (*n* = 539)	GG	CG	CC	G	C	0.204
		0.358 (*n* = 193)	0.458 (*n* = 247)	0.184 (*n* = 99)	0.587	0.413	

**Table 3:** Associations of the SNP genotypes with laying traits in the Jining Bairi chicken.

Site	Traits	Genotype			*p*-Value
g.−3015		GG (*n* = 193)	CG (*n* = 247)	CC (*n* = 99)	
	AFE	149.763 + 0.411	150.518 + 0.409	150.235 + 0.764	0.473
	E30	111.602 ± 1.690	113.539 ± 1.074	113.083 ± 1.346	0.411
	E50	137.520 ± 2.039	140.223 ± 1.293	138.818 ± 1.702	0.075
	CLFE	4.005 ± 0.052 ^a^	4.080 ± 0.048 ^ab^	4.224 ± 0.080 ^b^	0.019

AFE: age of first egg; E30: egg number at 30 weeks of age; E50: egg number at 50 weeks of age; CLFE: comb length at the age of the first egg. In the same line, different superscripts lowercase letters (^a, b^) means significant differences at *p* < 0.05.

## Data Availability

The data presented in this study are available on request from the corresponding author.
